# Constitutive Cyclin O deficiency results in penetrant hydrocephalus, impaired growth and infertility

**DOI:** 10.18632/oncotarget.21818

**Published:** 2017-10-12

**Authors:** Marc Núnez-Ollé, Carole Jung, Berta Terré, Norman A. Balsiger, Cristina Plata, Ramon Roset, Carlos Pardo-Pastor, Marta Garrido, Santiago Rojas, Francesc Alameda, Josep Lloreta, Juan Martín-Caballero, Juana M. Flores, Travis H. Stracker, Miguel A. Valverde, Francisco J. Muñoz, Gabriel Gil-Gómez

**Affiliations:** ^1^ Apoptosis Signalling Group, IMIM (Institut Hospital del Mar d’Investigacions Mèdiques), Barcelona, Spain; ^2^ Laboratory of Molecular Physiology, Universitat Pompeu Fabra, Barcelona, Spain; ^3^ Institute for Research in Biomedicine (IRB Barcelona), Barcelona Institute of Science and Technology, Barcelona, Spain; ^4^ Unit of Human Anatomy and Embryology, Department of Morphological Science, Faculty of Medicine, Universitat Autònoma de Barcelona, Bellaterra, Spain; ^5^ Servei de Patologia, Hospital del Mar-IMIM (Institut Hospital del Mar d’Investigacions Mèdiques), Barcelona, Spain; ^6^ Parc de Recerca Biomèdica de Barcelona, Barcelona, Spain; ^7^ Departamento de Medicina y Cirugía Animal, Facultad Veterinaria, Universidad Complutense de Madrid, Madrid, Spain

**Keywords:** Cyclin O, ciliogenesis, neurogenesis, hydrocephalus, development, Gerotarget

## Abstract

Cyclin O (encoded by *CCNO*) is a member of the cyclin family with regulatory functions in ciliogenesis and apoptosis. Homozygous *CCNO* mutations have been identified in human patients with Reduced Generation of Multiple Motile Cilia (RGMC) and conditional inactivation of *Ccno* in the mouse recapitulates some of the pathologies associated with the human disease. These include defects in the development of motile cilia and hydrocephalus. To further investigate the functions of Ccno *in vivo*, we have generated a new mouse model characterized by the constitutive loss of *Ccno* in all tissues and followed a cohort during ageing. *Ccno*^*-/-*^ mice were growth impaired and developed hydrocephalus with high penetrance. In addition, some *Ccno*^*+/-*^ mice also developed hydrocephalus and affected *Ccno*^*-/-*^ and *Ccno*^*+/-*^ mice exhibited additional CNS defects including cortical thinning and hippocampal abnormalities. In addition to the CNS defects, both male and female *Ccno*^*-/-*^ mice were infertile and female mice exhibited few motile cilia in the oviduct. Our results further establish *CCNO* as an important gene for normal development and suggest that heterozygous CCNO mutations could underlie hydrocephalus or diminished fertility in some human patients.

## INTRODUCTION

Cyclins are a family of proteins that share a common structural motif (the cyclin box) and fulfill diverse roles in the cell. These include cell cycle regulation (Cyclins A-E), transcriptional control (Cyclins H, K, T, L, etc.), splicing (Cyclin L) or the regulation of protein stability (Cyclin F). However, the function of a number of cyclin family members remains unknown (reviewed in [1]).

Cyclin O (CCNO) was previously identified in a search for cyclins able to activate Cdk2 during DNA damage and glucocorticoid-induced apoptosis. *Ccno* was induced very early in the process of apoptosis triggered by intrinsic stimuli, preceding the changes in the plasma membrane, mitochondrial dysfunction and the activation of caspases [2, 3]. Although the tight regulation of the *Ccno* gene by DNA damage and its role in apoptosis were demonstrated, the molecular targets of CCNO that are important for apoptosis remain unknown.

Recently, *CCNO* mutations were identified in a subset of Primary Ciliary Dyskinesia (PCD) patients affected by recurrent upper and lower airway infections that lead to the development of bronchiectasis and respiratory distress syndrome, as well as hydrocephalus (∼10%) and reduced fertility [4-6]. This PCD subset has been termed Reduced Generation of Multiple Motile Cilia (RGMC) and it has been shown that RGMC is also caused by mutations in the *MCIDAS* gene that is adjacent to *CCNO* on chromosome 5q [7]. The cellular defects resulting from these mutations were characterized by the almost complete lack of motile cilia in multiciliated cells (MCCs) present in the epithelia that line the upper airways. This resulted in defective clearance of mucus and foreign particles, leading to recurrent infections, and the destruction of the lung parenchyma.

A conditional loss of-function model for murine *Ccno* showed that the mutant mice recapitulated the main cellular defects described in patients carrying *CCNO* mutations, and that lack of *Ccno* led to perinatal lethality due to the development of hydrocephalus [8]. The authors also demonstrated that CCNO was required for the correct formation, or potentially maintenance, of the deuterosome, a cytosolic structure responsible for the amplification of centrioles required to form the basal bodies necessary for the generation of the multiple motile cilia characteristic of MCCs [9]. Therefore, CCNO appears to regulate multiple aspects of the multiciliogenesis process, although its immediate targets and precise roles remain unknown.

The deuterosome-mediated multiplication of centrioles, followed by the generation and apical docking of basal bodies and ciliary axoneme elongation in MCCs, is dependent on a highly regulated transcriptional program. *Ccno* is strongly induced during early MCC differentiation and the gene is located in a chromosomal locus that appears dedicated to multiciliation, as it contains *Cdc20B*, *Mir449a/b/c* and *Mcidas* that have all been implicated in the process [4, 7, 10-12]. Mcidas, more commonly referred to as Multicilin, is a transcriptional activator that shares homology to both Geminin (encoded by *Gmnn*), a key regulator of DNA replication, and GEMC1 (encoded by *Gmnc*), that plays a similar role as Mcidas in activating the transcriptional cascade necessary for MCC development [13-16]. In *in vitro* cultures of MCCs, the loss of *Ccno* resulted in the dramatic upregulation of multiple genes involved in MCC differentiation, including Multicilin, suggesting that it may modulate transcriptional output that controls deuterosome formation and centriole amplification [8].

In this study we further characterize the role of Ccno in the regulation of multiciliogenesis using a new, constitutive loss of function mouse model to assess the phenotypic consequences of complete *Ccno* deficiency. We find that the hydrocephalus phenotype is highly penetrant in *Ccno* deficient mice, in contrast to previous reports of conditional knockout mice [8] or what has been observed in human RGMC patients [4-6]. In addition, we find that some *Ccno* heterozygous mice are also affected, indicating partial haploinsufficiency of *Ccno*. Mice with hydrocephalus exhibited additional CNS defects, that were associated with the degree of severity of the hydrocephalus, and *Ccno*^*-/-*^ mice, exhibited male and female infertility and retarded growth rates. Our results suggest that residual activity of *CCNO* or genetic modifiers in some of the RGMC patient alleles identified may explain the incomplete penetrance of hydrocephalus and that heterozygous alleles of *CCNO* may cause pathologies in some human carriers of severe loss of function alleles.

## RESULTS

### Hydrocephalus and reduced survival in mice lacking *Ccno*

**Figure 1:**
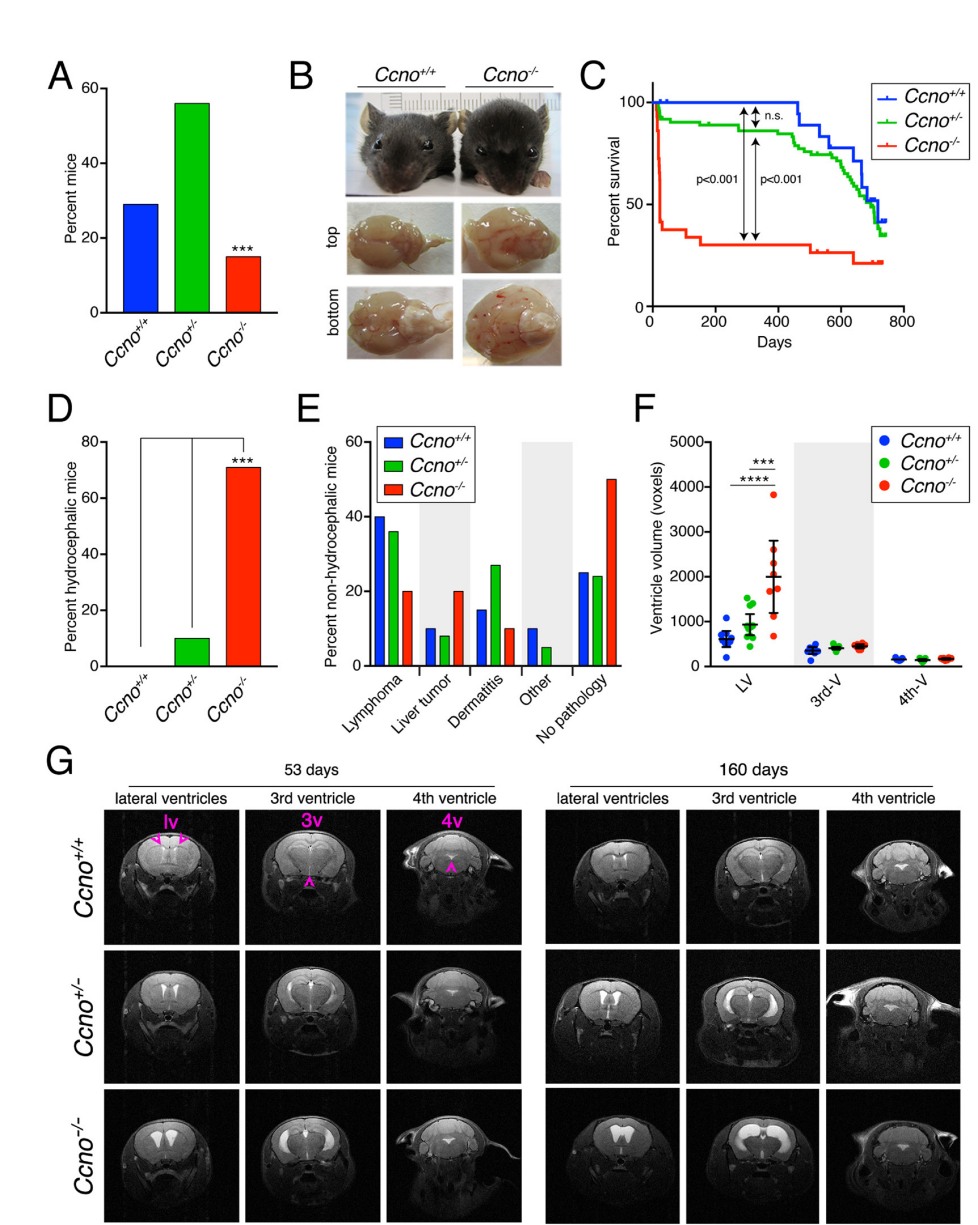
*Ccno* mutant mice develop hydrocephalus with high penetrance **A**. Genotype distribution of the offspring derived from *Ccno*^*+/-*^ matings (n=128). The distribution of genotypes differs from that expected by normal Mendelian inheritance ( p<0.0005, *chi square* test). **B**. Characteristic morphology of healthy *Ccno*^*+/+*^ and hydrocephalic *Ccno*^*-/-*^ P23 littermates. The top and bottom view of the *Ccno*^*-/-*^ brain shows generalized swelling due to the accumulation of cerebrospinal fluid. **C**. Kaplan-Meier survival plot of *Ccno*^*+/+*^, *Ccno*^*+/-*^ and *Ccno*^*-/-*^ mice. Littermates of mixed genotypes were kept in the same cage with free access to food and water and were sacrificed when moribund. The p-values were calculated using the log rank (Mantel-Cox) test. **D**. Incidence of hydrocephalus (percentage) in the cohort of mice used for the survival experiment (n=128; p<0.0005, one-way ANOVA with Bonferroni post hoc test). **E**. Pathologies found upon macroscopic examination in non-hydrocephalic mice (n=20 *Ccno*^*+/+*^, n=66 *Ccno*^*+/-*^, n=10 *Ccno*^*-/-*^). Differences among genotypes were not statistically significant by one-way ANOVA with Bonferroni post hoc test. **F**. Quantification of the volumes of the lateral (LV), third (3^rd^-V) and fourth (4^th^-V) brain ventricles of *Ccno*^*+/+*^ (n=9), *Ccno*^*+/-*^ (n=11) and *Ccno*^*-/-*^ (n=11) mice from the MRI images. Statistical analysis was done by one-way ANOVA with Tukey post hoc test. **G**. Representative examples of MRI images quantified in F. Young (upper left panels, mean age=53 days) and adult (upper right panels, mean age=160 days) *Ccno*^*+/+*^ (upper panels), *Ccno*^*+/-*^ (middle panels) and *Ccno*^*-/-*^ (lower panels) mice were analyzed. The location of the lateral (lv), third (3v) and fourth (4v) ventricles in the MRI images is indicated with purple arrowheads.

To investigate the impact of CCNO loss on murine development and ageing, we generated a constitutive *Ccno* loss of function model (Supplementary Figure 1) using two independent ES cell clones obtained from the KOMP Repository (www.komp.org). Two independent mouse lines were obtained that showed indistinguishable phenotypes and were used indistinctively. Constitutive *Ccno*^*-/-*^ mice were viable, but born at sub-Mendelian frequency (*chi square* test, p<0.0005; Figure 1A). About 70% of *Ccno*^*-/-*^ mice had to be sacrificed within the first month of postnatal life due to the development of severe hydrocephalus (Figure 1B, 1C and 1D). Mice developing early hydrocephalus showed a typically enlarged head and swollen brain (Figure 1B). However, *Ccno*^*-/-*^ mice surviving the early postnatal period (5-6 weeks) lived as long as *Ccno*^*+/+*^ and *Ccno*^*+/-*^ siblings (Figure 1C) without overt neurological consequences.

A small percentage of *Ccno*^*+/-*^ mice also developed early hydrocephalus (Figure 1D) and they had an overall trend towards reduced survival, but this did not reach statistical significance (Figure 1C). After the first postnatal month, lifespan was similar among the genotypes (Figure 1C) and macroscopic examination during necropsy did not reveal the occurrence of specific pathologies associated with any of the genotypes (Figure 1E). Monitored mice presented mainly with neoplasias (lymphomas and liver tumors) and ulcerative dermatitis, in agreement with data described for the C57BL/6 strain. It is reported that a low (1-3%) percentage of C57BL/6 mice develop spontaneous hydrocephalus (http://www.informatics.jax.org/external/festing/mouse/docs/C57BL.shtml). However, no hydrocephalic *Ccno*^*+/+*^ mice were observed in our cohorts, and in concordance with observations from human CCNO deficient patients, no *situs inversus* was observed in any of the mice analyzed [4, 5].

Magnetic Resonance Imaging (MRI) analysis revealed that *Ccno*^*-/-*^ mice developed symmetric, communicating (non-obstructive) hydrocephalus affecting mainly the lateral ventricles (LVs) of the brain (Figure 1F and 1G). The penetrance of hydrocephalus was nearly complete, already present in the *Ccno*^*-/-*^ mice analyzed at postnatal day 53 (Figure 1F and 1G). In addition, we observed several *Ccno*^*+/-*^ mice with different degrees of hydrocephalus in the LVs, indicating partial haploinsufficiency (Figure 1F, 1G and Supplementary Figure 2). Despite this, we did not observe any overt neurological defects in young or old mice monitored in our cohort.

### Severe defects in ependymal cilia formation in *Ccno* deficient mice

**Figure 2:**
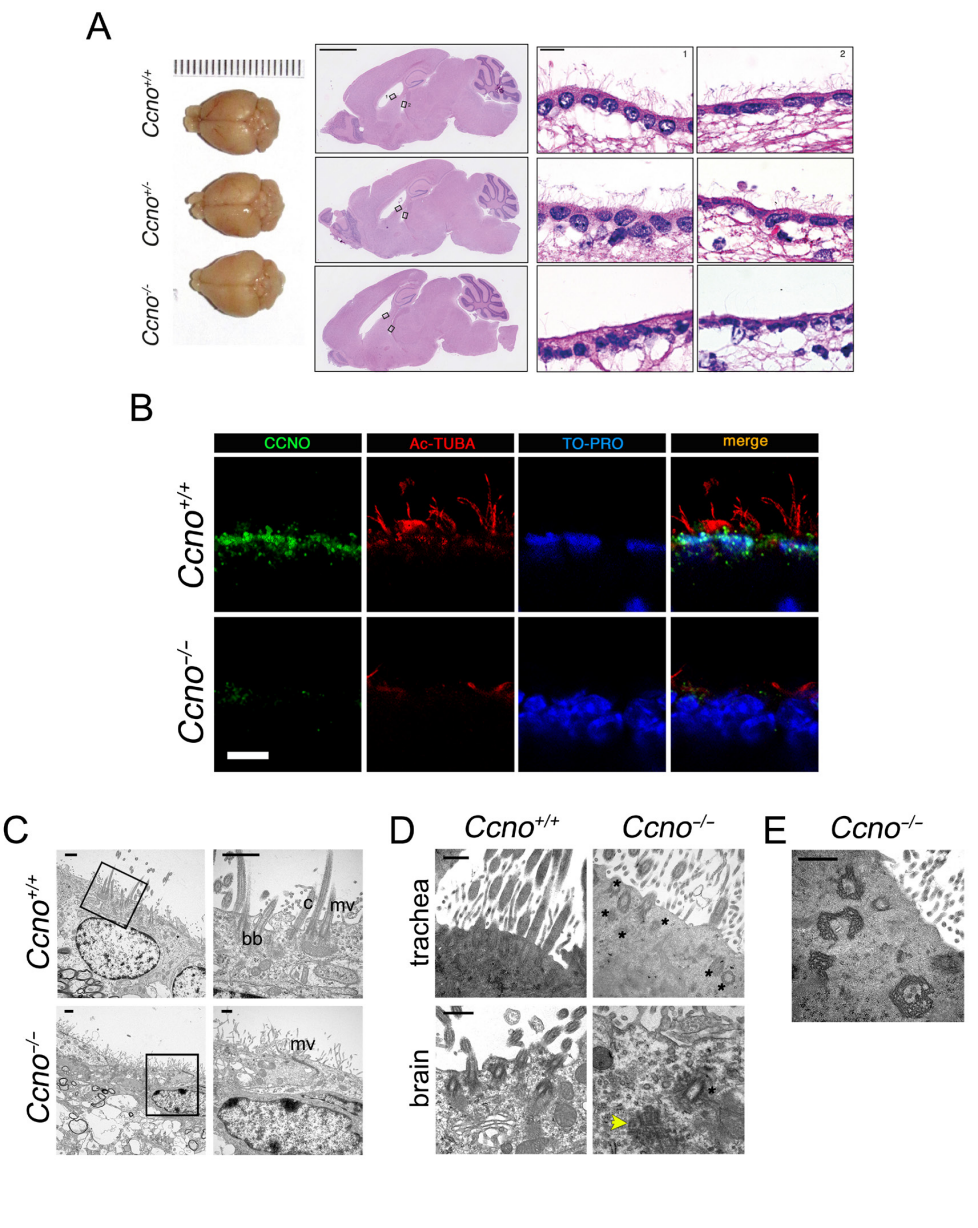
Loss of multiciliated cells in the ependymal and tracheal epithelia of *Ccno*^-/-^ mice A. Top view of the brain (left panels), sagittal sections (middle panels, H&E staining) and magnifications from the areas indicated by black rectangles (lateral and third ventricles, right panels, H&E staining) from *Ccno*^*+/+*^ (upper panels), *Ccno*^*+/-*^ (middle panels) and *Ccno*^*-/-*^ mice (lower panels). Scale bars = 2.5 mm (left panel) and 20 µm (right panels). **B**. Complete loss of immunoreactivity in *Ccno*^-/-^ ependymal cells. Confocal microscopy images of formaldehyde-fixed, paraffin embedded sections of brain from *Ccno*^+/+^ (upper panels) and *Ccno*^-/-^ (lower panels) adult mice were stained with antibodies against CCNO (green) and acetylated-α-Tubulin (red) and nuclei were counterstained with TO-PRO-3 (pseudocolored blue). Scale bar = 10 µm. **C**. Representative image of the ependymal epithelia from *Ccno*^+/+^ (upper panels) or *Ccno*^-/-^ (lower panels) by TEM. Ciliated and non-ciliated cells are seen in *Ccno*^+/+^ sections, with cilia (c), basal bodies (bb) and microvilli (mv) becoming apparent in higher magnification views (square regions). In contrast, few or no cilia are seen in *Ccno*^-/-^ cells and they are replaced by more abundant microvilli (mv). sc, secretory cells. Scale bars = 1 µm. **D**. Undocked, morphologically aberrant basal bodies (marked with asterisks) are seen in high magnification views of tracheal (upper panels) and brain (lower panels) sections of *Ccno*^-/-^ mice. A frayed, greatly altered basal body in the ependyma of *Ccno*^-/-^ mice is indicated with a yellow arrowhead. Scale bars = 500 nm. **E**. Accumulation of aggregates of modified microtubules are detected in tracheal sections of *Ccno*^-/-^ mice, most likely corresponding to dysmorphic deuterosomes [8]. Scale bar = 500 nm.

Similar to the conditional loss of function *Ccno* model [8], few cilia were observed in the ependymal epithelium of constitutive *Ccno*^*-/-*^ mice (Figure 2A and 2B) and CCNO was not detectable (Figure 2B). The drastic reduction in the number of ciliated cells was further confirmed by Transmission Electron Microscopy (TEM) (Figure 2C). In *Ccno*^*-/-*^ mice, cells that would have normally become MCCs were characterized by long, abundant microvilli, aberrant basal bodies (Figure 2D) and modified microtubule structures that corresponded to dysmorphic deuterosomes (Figure 2E). The higher percentage of mice developing hydrocephaly, as well as the nearly complete absence of cilia in the ependymal epithelium (Figure 2A and 2B), suggest that the constitutive deletion of *Ccno* leads to a more penetrant phenotype than what has been observed previously in mice with a conditional loss of function allele [8].

### Cortical and hippocampal abnormalities and neuronal cell damage in hydrocephalic *Ccno*^-/-^ mice

**Figure 3:**
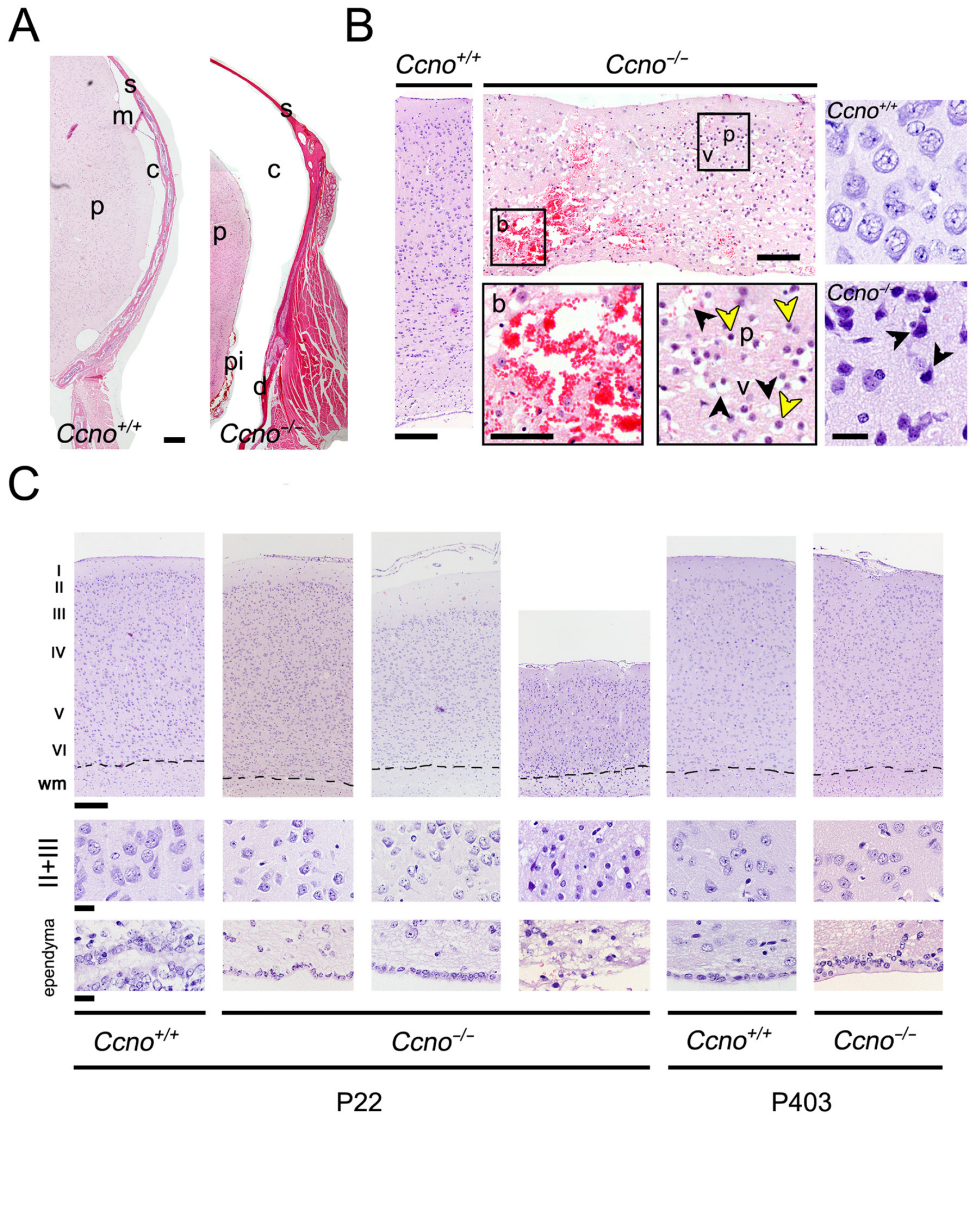
Neuronal damage in the CNS of *Ccno*^-/-^ mice **A**. Hydrocephalic *Ccno*^*-/-*^ mice developed subdural hygroma. p: brain parenchyma; s: skull; m: meninges; c: cerebrospinal fluid; d: dural membrane; pi: pia mater. Scale bar = 200 µm. **B**. Intraparenchymal hemorrhage in a hydrocephalic P23 *Ccno*^*-/-*^ mouse (central upper panel); in the left panel an image of the brain cortex of a P23 *Ccno*^*+/+*^ control mouse is shown. Bars = 200µm. Magnifications of the boxed areas containing the bleeding (b) and the neurons with pyknotic nuclei (p, yellow arrowheads) and vacuolated parenchyma (v, black arrowheads) are shown in the central lower panels. Scale bar = 100µm. The lower right panel shows acidophilic pyramidal neurons (black arrowheads) in the cortex of a P23 *Ccno*^*-/-*^ mouse. The corresponding *Ccno*^*+/+*^ control is shown in the upper right panel. Scale bar = 20 µm. **C**. Cortical thinning in the brain of *Ccno*^*-/-*^ mice. The layered structure present in *Ccno*^*+/+*^ and non-hydrocephalic P22 *Ccno*^*-/-*^ mice (left image) is altered in hydrocephalic (middle image) and severely affected in highly hydrocephalic, moribund P22 *Ccno*^*-/-*^ mice (right image). Acidophilic neurons and parenchyma vacuolation can be appreciated. The cortical layers are comparable in aged (P403) *Ccno*^*+/+*^ and *Ccno*^*-/-*^ non-hydrocephalic littermates (right panels). Scale bar = 200 µm. A magnification of the neurons from the II and III layers is shown in the middle panels of the Figure. The innermost end of the cortical images containing the ependyma is shown in the lower panels. Scale bars = 20 µm. wm: White matter.

The development of severe hydrocephalus in *Ccno*^*-/-*^ mice led to profound morphological changes in the anatomy and histology of the CNS. Subdural hygroma, an accumulation of cerebrospinal fluid (CSF) without blood in between the dura mater and the brain parenchyma, was observed (Figure 3A). Other signs indicative of CNS damage such as intraparenchymal hemorrhages, tissue vacuolation, pyknotic nucleus and acidophilic neurons were observed in hydrocephalic *Ccno*^*-/-*^ animals (Figure 3B). We also observed that the cortex was considerably thinner and the ependymal layer lost in severely affected *Ccno*^*-/-*^ mice. However, we found that in both young (P22) and aged (P403) *Ccno*^*-/-*^ animals that were not overtly hydrocephalic, the structure of the cortex layers was similar to that of *Ccno*^*+/+*^ littermates (Figure 3C). The lack of CCNO also did not result in the disappearance of the ependyma. In both young (P16) and adult (P84) *Ccno*^*-/-*^ mice, the brain ventricles were covered by a monolayer of cells that had few cilia (marked by acetylated-α-Tubulin) compared to *Ccno*^*+/+*^ mice, but expressed comparable levels of the ciliated lineage transcription factor FOXJ1, the crucial multiciliogenesis regulator TP73 [17] and were postmitotic, as determined by the lack of expression of MKi67 (Supplementary Figure 3). These data indicate that the multiciliated cell lineage is generated in *Ccno*^*-/-*^ mice but they do not assemble mature cilia.

**Figure 4:**
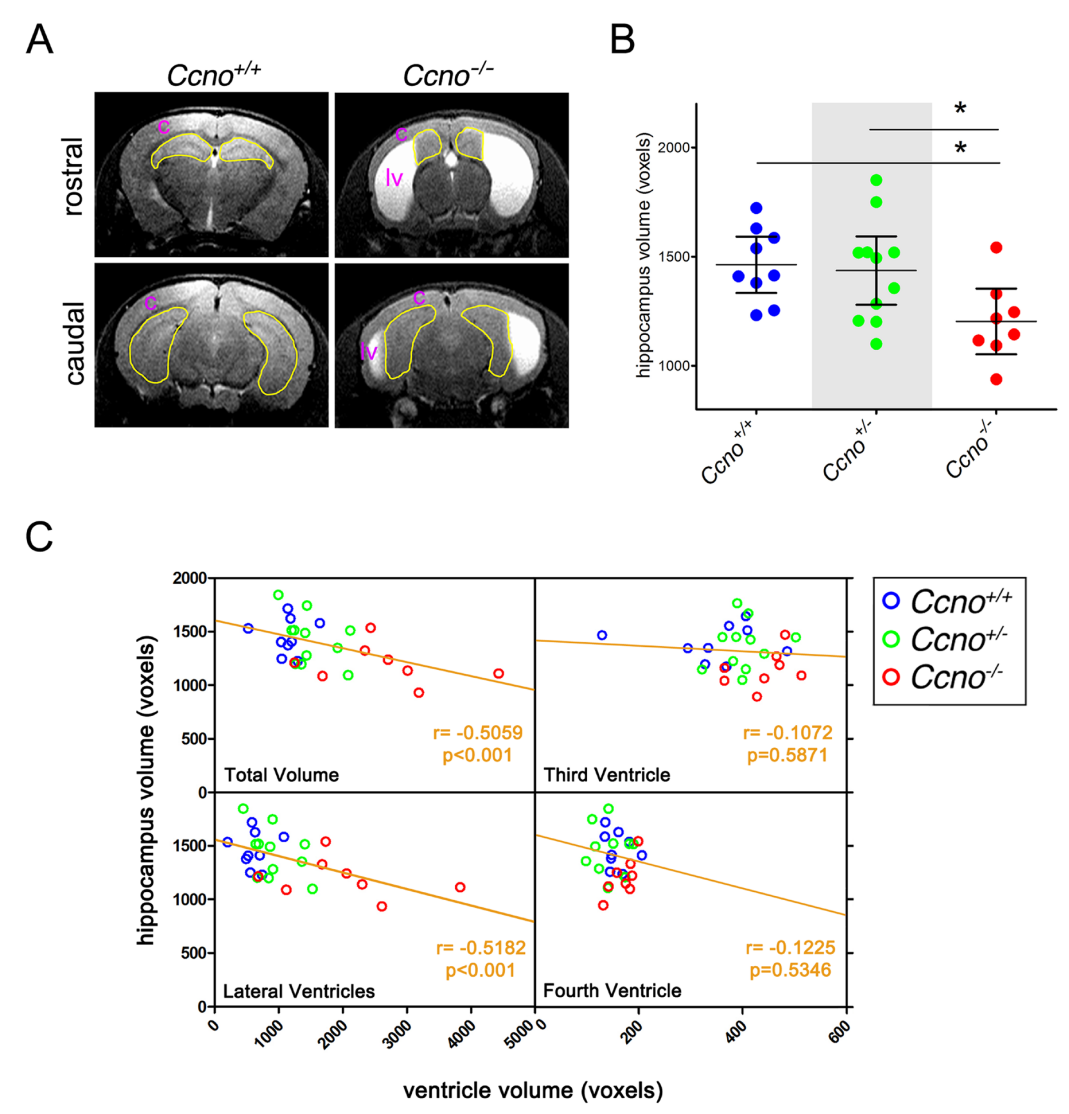
Hippocampal abnormalities in *Ccno* mutant mice **A**. Representative MRI images of the hippocampi quantified in B. The areas delimited in yellow correspond to the hippocampal area quantified. c: brain cortex; lv: lateral ventricle. **B**. Quantification of the volumes of the hippocampi of *Ccno*^*+/+*^ (n=9), *Ccno*^*+/-*^ (n=11) and *Ccno*^*-/-*^ (n=11) mice from the MRI images. Statistical analysis was done by one-way ANOVA with the Tukey post hoc test. **C**. Correlation plots between the volume of the ventricles and the volume of the hippocampus quantified from the MRI images. A significant Pearson correlation test was obtained between the total volume of the ventricles or the volume of the lateral ventricles and the volume of the hippocampus.

In addition to the cortical abnormalities, reduced hippocampal volume was also observed in *Ccno*^*-/-*^ mice. The volume of the hippocampus of the mice analyzed in Figure 1F was measured through the analysis of the Magnetic Resonance Imaging (MRI) images (Figure 4A). Quantification of the MRI images revealed that the volume of the hippocampi of *Ccno*^*-/-*^ mice was significantly smaller than those of *Ccno*^*+/+*^ or *Ccno*^*+/-*^ mice (Figure 4B). The volume of the hippocampi, showed an inverse correlation with the volume of the lateral but not the third or fourth ventricles of the same group of mice (Figure 4C), indicating that the higher the degree of hydrocephalus, the smaller the volume of the hippocampus, that is the brain center for learning and memory.

Collectively, our findings suggest that the alterations observed in ependymal cells caused by reduced levels of CCNO may reduce the reabsorption of CSF through a trans-ependymal pathway leading to the development of hydrocephalus. The alterations observed in the cortex and the reduction of the volume of the hippocampus are likely secondary effects of severe hydrocephalus, as there is a strong inverse correlation between the volume of the lateral ventricles and hippocampal size. These results are in line with published data [8] and our observations that *Ccno* expression is not detectable in the developing mouse brain cortex and hippocampus.

### Impaired growth and infertility in *Ccno*^-/-^ mice

**Figure 5:**
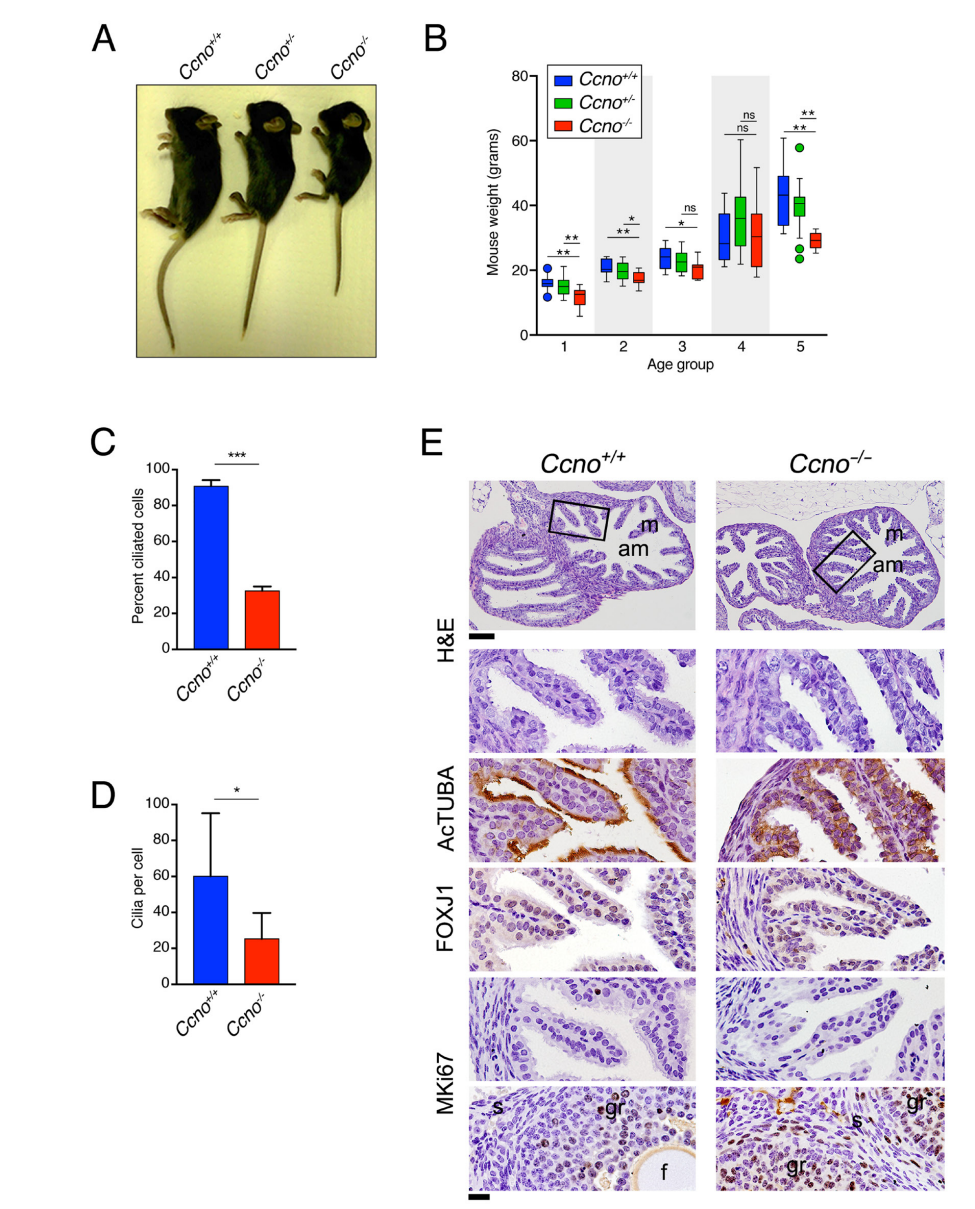
Impaired growth and infertility of *Ccno* mutant mice **A**. Representative image of *Ccno*^*+/+*^, *Ccno*^*+/-*^, and *Ccno*^*-/-*^ littermates at P30. **B**. Weights of *Ccno*^*+/+*^ (blue bars), *Ccno*^*+/-*^ (green bars) and *Ccno*^*-/-*^ mice (red bars) from 21 to 742 days postpartum. Age group 1: P21-P34; Age group2: P35-P49; Age group 3: P50-P99; Age group 4: P100-P499; Age group 5: P500-P742. Circles represent outliers. A minimum of five animals were plotted for each age group. Statistical significance was determined by the unpaired two-way Mann–Whitney *U* test. **C** and **D**. Quantification of the number of ciliated cells (C) and the number of cilia per cell (D). Statistical significance was determined by the unpaired *Student’s t*-test. **E**. Histochemical analysis of the oviductal epithelium reveals few MCCs but most of the cells of the epithelium expressing the MCC lineage marker FOXJ1 and are negative for MKi67 in young (P129) *Ccno*^*-/-*^ mice. A positive control for MKi67 (granulosa cells from the ovary) are shown in the bottom panels. m: mucosa; am: ampulla; s: stroma; gr: granulosa; f: follicle. Scale bars = 100µm (top panels) and 20µm (rest of the panels).

In addition to the CNS abnormalities, we observed that loss of *Ccno* led to growth defects. *Ccno*^*-/-*^ mice were runted and showed significantly lower weight gain than *Ccno*^*+/+*^ or *Ccno*^*+/-*^ mice until about 4 months of age (Figure 5A and 5B). *Ccno*^*+/-*^ mice showed a trend towards an intermediate size but were not statistically different than wild type animals (Figure 5B). It was previously reported that both male and female *Ccno*^*-/-*^ mice were fertile [8]. However, the constitutive loss of *Ccno* in our model led to apparent infertility in both males (36 pups from 2 *Ccno*^*+/+*^ male x female crosses; 0 pups from 3 *Ccno*^*-/-*^ male x *Ccno*^*+/+*^ female crosses) and females (100 pups from 7 *Ccno*^*+/+*^ male x female crosses; 0 pups from 3 *Ccno*^*+/+*^ male x *Ccno*^*-/-*^ female crosses). Consistent with this, we observed few cilia in the oviducts of female *Ccno*^*-/-*^ mice, similar to what was previously observed in the infertile *Gemc1*^*-/-*^ mice (Figure 5C), and the multiciliated cells had significantly lower numbers of cilia per cell (Figure 5D). Similar to what we observed in the ependyma, the folds of the oviduct in *Ccno*^*-/-*^ mice were covered by cells expressing low levels of acetylated-α-Tubulin compared to *Ccno*^*+/+*^ mice but expressed comparable levels of the ciliated lineage transcription factor FOXJ1 and were postmitotic, as determined by the lack of MKi67 expression (Figure 5E). These data show that the multiciliated cell lineage is present in the oviducts of *Ccno*^*-/-*^ mice but they have a strongly reduced number of cilia.

Despite the striking reduction of cilia in the MCCs of the ependyma, oviduct and airway, no obvious signs of chronic infections or mucus accumulation were observed upon histological analysis of the lungs of young or old *Ccno*^*-/-*^ mice. This is likely due to the strict Specific Pathogen Free (SPF) housing conditions and is similar to what we previously observed with *Gemc1*^*-/-*^ mice that completely lack MCCs in the airway and did not show increased morbidity in the same housing facility [15].

## DISCUSSION

Here we have described a new mouse model characterized by the constitutive loss of *Ccno* in all tissues. Although, we observed that our *Ccno*^*-/-*^ mice were born at near-Mendelian frequency, they developed hydrocephalus with a higher penetrance than previously reported for conditional *Ccno* knockout mice [8] or human RGMC patients [4, 5]. During the first month of postnatal development, around 70% of *Ccno*^*-/-*^ mice developed severe communicating hydrocephalus due to the accumulation of cerebrospinal fluid in the lateral ventricles of the brain (Figure 1B, 1D, 1F and 1G). However, *Ccno*^*-/-*^ mice surviving the early postnatal period (5-6 weeks) lived as long as *Ccno*^*+/+*^ and *Ccno*^*+/-*^ siblings (Figure 1C), most likely due to compensation of the hydrocephalus through the thinning of the brain parenchyma. We also observed that pups developing hydrocephalus during the lactation period were usually eaten by their mothers, potentially explaining the lower frequency of homozygous conditional mice observed at the time of genotyping (P21) in previous studies [8].

Additionally we observed that about 10% of the heterozygous mice developed overt signs of hydrocephalus (Figure 1D) within the first month of life and some of the mice analyzed as early as day P53 showed enlarged ventricles in the MRI images (Figure 1F and 1G, Supplementary Figure 2). While this phenotype was not as penetrant in *Ccno*^*+/-*^ compared to *Ccno*^*-/-*^ mice, it should be considered in the context of human chronic hydrocephalus, as many families carry apparent loss of function alleles of *CCNO*, that has so far emerged as the most commonly mutated gene in RGMC [5]. Some neurological signs of ciliopathy, such as hydrocephalus, may remain underdiagnosed because of the late manifestation of the clinical indicators.

One example is Normal Pressure Hydrocephalus (NPH), a form of communicating hydrocephalus that can progress over decades without severe neurological manifestations. Idiopathic NPH is a relatively common disease of the elderly, having an estimated prevalence of 5.9% in people aged 80 and older [18] and it is very likely that it is underdiagnosed, and therefore greatly underestimated, due to the lack of consistent clinical symptoms [19]. As the molecular cause of human NPH is unknown, it is tempting to speculate that *CCNO* haploinsufficiency could represent an unidentified cause for idiopathic NPH.

In addition to hydrocephalus, we found numerous indications of severe neuronal damage in cortex of hydrocephalic *Ccno*^*-/-*^ mice, including intraparenchymal hemorrhage, pyknotic nuclei, tissue vacuolation and acidophilic neurons (Figure 3), likely the result of intracranial pressure from hydrocephalus. The development of hydrocephalus also appeared to affect the hippocampus, as the volume of this structure inversely correlated with the volume of the lateral ventricles. In MRI images it can be appreciated that the enlarged lateral ventricles distorted the normal appearance of the neighboring hippocampus, suggesting that high intracranial pressure is the cause of the smaller size of this structure in *Ccno*^*-/-*^ mice.

Besides the defects we observed in the CNS, *Ccno*^*-/-*^ mice were growth impaired and it is likely that hydrocephalus is also responsible for the lower weight of mice younger than 34 days. Weaning hydrocephalic mice have impaired access to mother’s milk and became cachectic. Those that were not eaten by their mother also showed problems feeding after weaning and were clearly runted until they were older than 3 months (Figure 5B). The differences in older mice were less pronounced but most *Ccno*^*-/-*^ mice weighed less than their wild type littermates. This could reflect roles for CCNO in the development or maintenance of adult tissues or be secondary to neurological deficits resulting from penetrant hydrocephalus. *Ccno*^*+/-*^ mice showed an overall trend towards intermediate weights that may reflect the fact that a subset of these animals developed hydrocephalus (Figure 1D, 1F and 1G).

At odds with previous reports in conditional knockout mice [8], we found that our *Ccno*^*-/-*^ mice were infertile. Infertility has been reported in male and female (22%) RGMC patients [4, 5] but the full impact of *CCNO* on fertility in RGMC remains unclear due to the rarity of the disease. The almost complete lack of MCCs in the oviduct of *Ccno*^*-/-*^ mice (Figure 5C-5E) is similar to what was observed in the infertile *Gemc1*^*-/-*^ females [15] and suggests that an inability to properly mobilize gametes may underlie the phenotype. We can detect the expression of *CCNO* in the cytoplasm of MCCs in human Fallopian tubes (Supplementary Figure 4) suggesting that its role in the MCCs of human oviducts is likely to be conserved. We are not sure why there is a discrepancy between the fertility phenotypes of our mice and those previously reported [8], but it has been described that some PCD patients with severe motile cilia dysfunction can spontaneously conceive, suggesting that fertility outcomes may be highly variable and influenced by some compensatory mechanisms [20]. Alternatively, incomplete deletion of the conditional *Ccno* allele in the germ cells may potentially explain the discrepancy.

Male infertility is also frequent amongst PCD patients (reviewed in [21]) and mouse models deficient for genes involved in the generation or function of motile cilia, such as *Poc1a* [22] or *Cfap54* [23], showed impaired spermatogenesis and male infertility. *Ccno* may play a direct role in the regulation of spermatogenesis, either through the regulation of DNA damage-induced apoptosis [3], that may influence the survival of the germinal cells during the process of meiosis, or through the regulation of transcription necessary for spermiogenesis [8]. A nonexclusive possibility is that the efferent ducts (EDs) of the epididymis [24], which contain MCCs that are necessary for the transit of immotile sperm, are not properly formed in *Ccno*^*-/-*^ mice. The male infertility observed in mice lacking both E2F4 and E2F5 has been proposed to result due to defects solely in the development of MCCs in the ED [25] and future work will be needed to fully characterize this defect in *Ccno*^*-/-*^ mice.

Overall, constitutive or conditional loss of *Ccno* in mice leads to clear defects in the generation of MCCs in multiple tissues and the development of pathologies consistent with what has been observed in RGMC patients, including hydrocephalus and infertility. We report here the further characterization of severe CNS defects that result as a consequence of hydrocephalus, as well as others that may be of independent origin following a reduction in *Ccno* levels. The increased penetrance of these phenotypes following constitutive *Ccno* loss, as well as the emergence of hydrocephalus and other defects in some heterozygous mice, suggests that some of the RGMC alleles identified may retain residual function. Moreover, it could indicate that haploinsufficiency of *CCNO* could underlie hydrocephalus or other defects in undiagnosed human patients. Further work will be needed to define the full range of CCNO functions, as well as its molecular targets.

## MATERIALS AND METHODS

### Animal samples

The research presented has been conducted in accordance with the ethical standards and according to national and international guidelines and has been approved by the authors’ institutional review board (the Departament d’Agricultura, Ramaderia, Pesca, Alimentació i Medi Natural of Generalitat de Catalunya).

### Human samples

The use of human samples were authorized by the Ethics Committee for Clinical Investigation of the Parc de Salut Mar, approval ID CEIC PSMAR: 2013/5274/I and obtained from the Parc de Salut MAR Biobank (MARBiobanc), reference 2015S028.

### Generation of *Ccno* knockout mice

Three independent ES cell clones carrying a substitution of the complete coding region of the mouse *Ccno* gene by the *E.coli lacZ* gene and a loxP-flanked *neo* cassette were obtained from the KOMP Repository (C57BL/6N-Ccno^tm1(KOMP)Vlcg^, Project KO-1978, UCLA, USA). The ES cell clones were injected into C57Bl/6J blastocysts at the CNIO’s Transgenic Mice Core Unit (CNIO, Madrid, Spain) and from two of the ES cell clones, six highly chimeric males were obtained. Chimeric males were crossed to C57BL/6J WT mice (Charles River Laboratories International, Inc.) and two lines corresponding to the original two ES cell clones were established and bred independently. No differences were observed between the two *Ccno*^*-/-*^ lines and were used indistinctively in the study. Both lines were crossed to the *Cre deleter* strain [26] to remove the *neo* selectable marker (*delta* allele, Supplementary Figure 1A). After segregation of the *Cre* transgene, the resulting lines showed to be indistinguishable from the parental. Both the *neo*-containing lines (KO[-] allele, Supplementary Figure 1A) or the delta (∆) allele bearing line were used indistinctively in the presented work. The mutations have been bred continuously in a pure C57BL/6J background. Mice were housed at all times under SPF conditions at the PRBB animal facility. Mice were genotyped by PCR at the time of weaning.

### *Ccno*-deficient mouse genotyping

Mice were weaned at P21, a tail biopsy was taken and the genomic DNA isolated. To detect *Ccno*-WT and the *Ccno*-KO (-) alleles, a common primer (SD: 5’- GGAACTCAGCCTCCTGACTG -3’) was used. The antisense primers used were specific for the *Ccno* wild-type allele (E3F1: 5’- GCTGAGCCTAACGGATTACG-3’) and for the *neo* cassette (NeoF: 5’- TCATTCTCAGTATTGTTTTGCC -3’). To genotype the delta (∆) allele, independent PCR reactions were assembled for each allele. The WT allele was detected with the primers SD and E3F1 and the KO (-) allele with the primers LacInF (5’- GGTAAACTGGCTCGGATTAGGG-3’) and LacInR (5’- TTGACTGTAGCGGCTGATGTTG -3’). All the primers for PCR genotyping were used at 0.5 µM final concentration. 10X PCR buffer: 160mM (NH_4_)_2_SO_4_, 670 mM Tris-HCL (pH 8.8), 2 mM dNTPs, 30 mM MgCl_2_ and 0.7U BIOTAQ (Bioline, Germany).

Thermocycling: step 1, 2 min at 93°C; step 2, 40 cycles of 30 seconds at 93°C, 30 seconds at 55°C and an extension of 50 seconds and one cycle of 10 minutes at 72°C. The DNA products were 652 and 471 bp (for *Ccno* WT and KO [-] alleles) and 210 bp for the delta (∆) allele.

### Survival analysis

Crosses of *Ccno*^*+/-*^ mice were set and the progeny genotyped and housed in standard conditions with full access to food and water. Because of the development of hydrocephalus before weaning, newborn mice were monitored twice a day until weaned. After weaning, the cohort (20 *Ccno*^*+/+*^, 73 *Ccno*^*+/-*^, 35 *Ccno*^*-/-*^) was monitored daily and the mice were sacrificed at any sign of disease. At day P742 all the surviving animals were sacrificed and necropsies carried out.

### Fertility analysis

Three crosses of 8-9 weeks-old virgin male or female *Ccno*^*-/-*^ mice with *Ccno*^*+/+*^ mates were set to check the fertility of male and female *Ccno*^*-/-*^ mice. As controls, age-matched, virgin *Ccno*^*+/+*^ crosses were set. Control *Ccno*^*+/+*^ and *Ccno*^*-/-*^ breedings were set the same day and the birth date and number of pups checked.

### Necropsy and pathological analysis

Tissue samples were fixed in 10% buffered formaldehyde, dehydrated in increasing concentrations of ethanol, embedded in paraffin wax, sectioned at 3µm, stained with hematoxylin and eosin (H&E), dehydrated and mounted. Images were taken with an Olympus BX61 microscope.

### Antibodies

Mouse monoclonal anti-acetylated α-TUBULIN (6-11B-1) was from Sigma-Aldrich and Santa-Cruz Biotechnology. The anti-ciliated cell marker LhS28 and anti-FOXJ1 antibodies were from Santa-Cruz Biotechnology. The anti-cell cycle marker Ki67 mouse monoclonal (clone B56) was from BD Biosciences. The anti-p73 rabbit monoclonal EP436Y was from Abcam. Mouse monoclonal antibody 7E8 (IgG1) directed against human CCNO was custom made by GenScript and purified by Protein G-Sepharose chromatography. The C2 antibody against mouse CCNO has been described previously [27]. Fluorescently labeled anti-mouse and anti-rabbit secondary antibodies were from Thermo-Fisher. EnVision^R^ anti-rabbit or anti-mouse system were from DakoCytomation.

### Immunohistochemistry and immunofluorescence

Immunohistochemical (IHC) and immunofluorescence (IF) analyses were performed using 3µm sections of paraformaldehyde-fixed, paraffin-embedded tissue blocks. Antigen retrieval was done by boiling the slides in 10 mM sodium citrate pH 6 for 10 minutes.

For IF, after deparaffination and antigen retrieval the slides were blocked in PBS containing 1% BSA and 5% horse serum for 30 minutes. Primary and secondary antibodies were diluted in PBS containing 1% BSA. Primary antibodies were incubated overnight at 4°C and secondary antibodies were incubated for 1 hour at room temperature. Slides were mounted with either with MOWIOL or Fluoromount G (SouthernBiotech) containing TO-PRO-3 (Thermo Fisher). Images were taken with Leica SP2 or Leica SP5 and SP8 confocal microscopes.

To compare samples from *Ccno*^+/+^ and *Ccno*^-/-^ mice, the negative control settings were established by staining each sample omitting the primary antibody. The positive control settings were determined using the sample from *Ccno*^+/+^ mice and the same settings were used to acquire the images corresponding to the sample from *Ccno*^-/-^ mice.

For IHC, slides were blocked with filtered 5% non fat milk dissolved in PBS. Primary and secondary antibodies were diluted in PBS containing 1% BSA. The primary antibody against was incubated for 90 min at 37°C or overnight at 4°C. As secondary antibody, EnVision^R^ anti-rabbit or anti-mouse system was applied (DakoCytomation). Sections were counterstained with hematoxylin, dehydrated and mounted. Images were taken with an Olimpus BX61 microscope.

### MRI imaging and cerebral ventricle volume quantification

Mice were scanned in a 7.0T BioSpec 70/30 horizontal animal scanner (Bruker BioSpin), equipped with a 12-cm inner diameter actively shielded gradient system (400 mT/m). The receiver coil was a phased array surface coil for mouse brain. Mice were placed in a supine position in a Plexiglas holder with a nose cone for anesthesia administration (isoflurane in a mixture of 30% O_2_ and 70% N_2_O) and maintained under controlled temperature during the acquisition period. Tripilot scans were carried out for accurate positioning of the animal’s head in the isocenter of the magnet. T2 relaxometry maps/weighted images were acquired with a multi-slice multi-echo acquisition sequence with 16 effective echo times from 11 to 176 ms, TR=4764 ms, FOV=20x20x6 mm3 , matrix size 256x256x24 pixels^3^ and spatial resolution 0.078x0.078x0.5 mm^3^ /pixel. Images were analyzed with MRIcro Software. Regions of interest (ROIs) for lateral, third and fourth ventricles as well for the hippocampus were manually drawn on axial images and the number of voxels of each ROI was calculated.

### Transmission electron microscopy

TEM was performed on brain and tracheal samples obtained by perfusion of the mice with a 2% paraformaldehyde/2% glutaraldehyde solution in 0.2M cacodylate buffer. Organs were extracted and stored in cacodylate buffer until further processing. After postfixation with 2% osmium tetroxide samples were dehydrated and embedded in Epon LX112 (Ladd Research Industries). Semi-thin sections (approximately 1µm thick) were cut and stained with toluidine blue. Ultrathin sections (60-80nm) were obtained with an ultramicrotome, placed on parlodion/carbon-coated nickel grids and stained with lead citrate and uranyl acetate. The grids were examined using a Phillips CM100 electron microscope.

### Statistical analysis

Results are presented either as mean values ± SEM or else the values for individual mice are plotted and the median value is shown. The Log-rank test (Mantel-Cox) was used to determine the statistics of animal survival. Where indicated, parametric one-way ANOVA test followed by a post-hoc Tukey or Bonferroni tests were applied. When not, non-parametric Kruskal-Wallis test was applied to all data to determine the equality of all genotypes. When the null hypothesis that all genotypes were equal was rejected with a p-value > 0.05, Mann–Whitney U tests were performed for all possible pairs of data and identified those genotypes that were significantly different, which are indicated in the figures. Pearson correlation coefficient was calculated to analyze the relation between the volumes of hippocampus and cerebral ventricles. For the statistical analysis, the IBM SPSS 20 statistical package (SPSS Inc, Chicago, IL) or GraphPad Prism 7 (GraphPad software Inc, San Diego, CA) was used. A p-value <0.05 was considered to be significant and the results represented as * p<0.05: ** p<0.005, *** p<0.0005 and ****p<0.0001. *ns* indicates that the difference between the two groups is not significant.

## SUPPLEMENTARY MATERIALS FIGURES



## Abbreviations

Ab: Antibody
BSA: Bovine Serum Albumin
CNS: Central Nervous System
CSF: Cerebrospinal Fluid
H&E: Hematoxilin and eosin
IF: Immunofluorescence
IHC: immunohistochemistry
KO: knockout
MCC: Multiciliated Cell
NPH: Normal Pressure Hydrocephalus
PBS: Phosphate Buffered Saline
PCD: Primary Ciliary Dyskinesia
RGMC: Reduced Generation of Multiple Motile Cilia
TEM: Transmission Electron Microscopy
SEM: Standard Error of the Mean
SPF: Specific Pathogen Free
WT: Wild Type
