# Supplementary material for: Constitutive Cyclin O deficiency results in penetrant hydrocephalus, impaired growth and infertility
Source: Oncotarget. 2017 Oct 12;8(59):99261–73. doi: 10.18632/oncotarget.21818 (PMC5725090; doi:10.18632/oncotarget.21818)
Supplement: Supplementary file 1 [file oncotarget-08-99261-s001.pdf]

Constitutive Cyclin O deficiency results in penetrant hydrocephalus, impaired growth and infertility

SUPPLEMENTARY MATERIALS

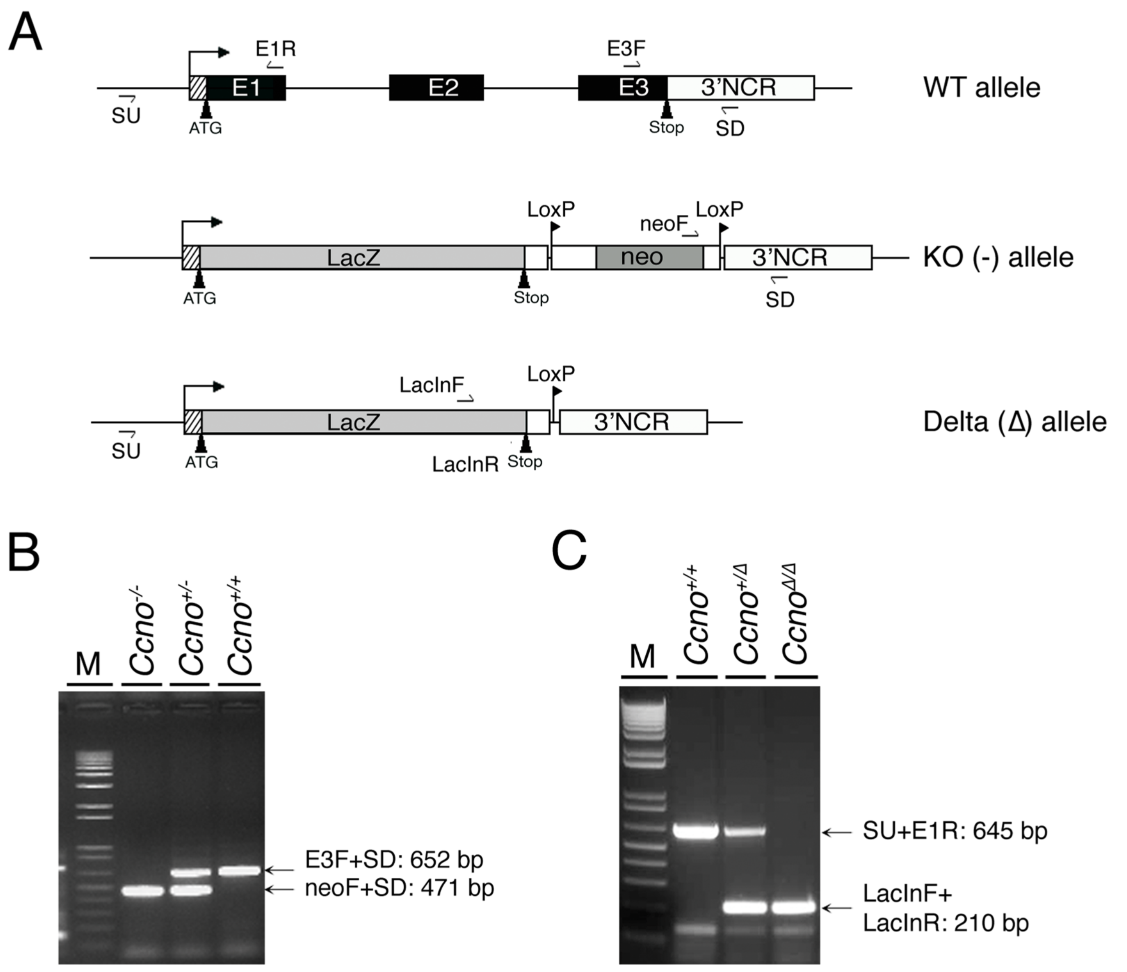

Supplementary Figure 1: Generation of Ccno knockout mice. A. Targeting of the *Ccno* locus. The two independently isolated ES cell clones AD9 and AC4 were obtained from the KOMP Repository (www.komp.org). Both clones carry a substitution of the coding region of the *Ccno* gene (WT allele) by the *E.coli LacZ* gene integrated in the exact translation starting site of the endogenous *Ccno* gene and followed by a loxP-flanked *neo* cassette (KO[-] allele). Chimeric males were crossed to C57BL/6J WT mice and two lines corresponding to the original two ES cell clones were established and bred independently (neo lines). By crossing to a Cre-*deleter* strain [26], the *neo* selectable marker was excised (delta [Δ] allele). Both neo lines and the delta line were used indistinctively in the study. B. PCR genotyping of the WT and KO (-) targeted *Ccno* alleles. C. PCR genotyping of the *Ccno* WT (-) and delta (Δ) alleles.

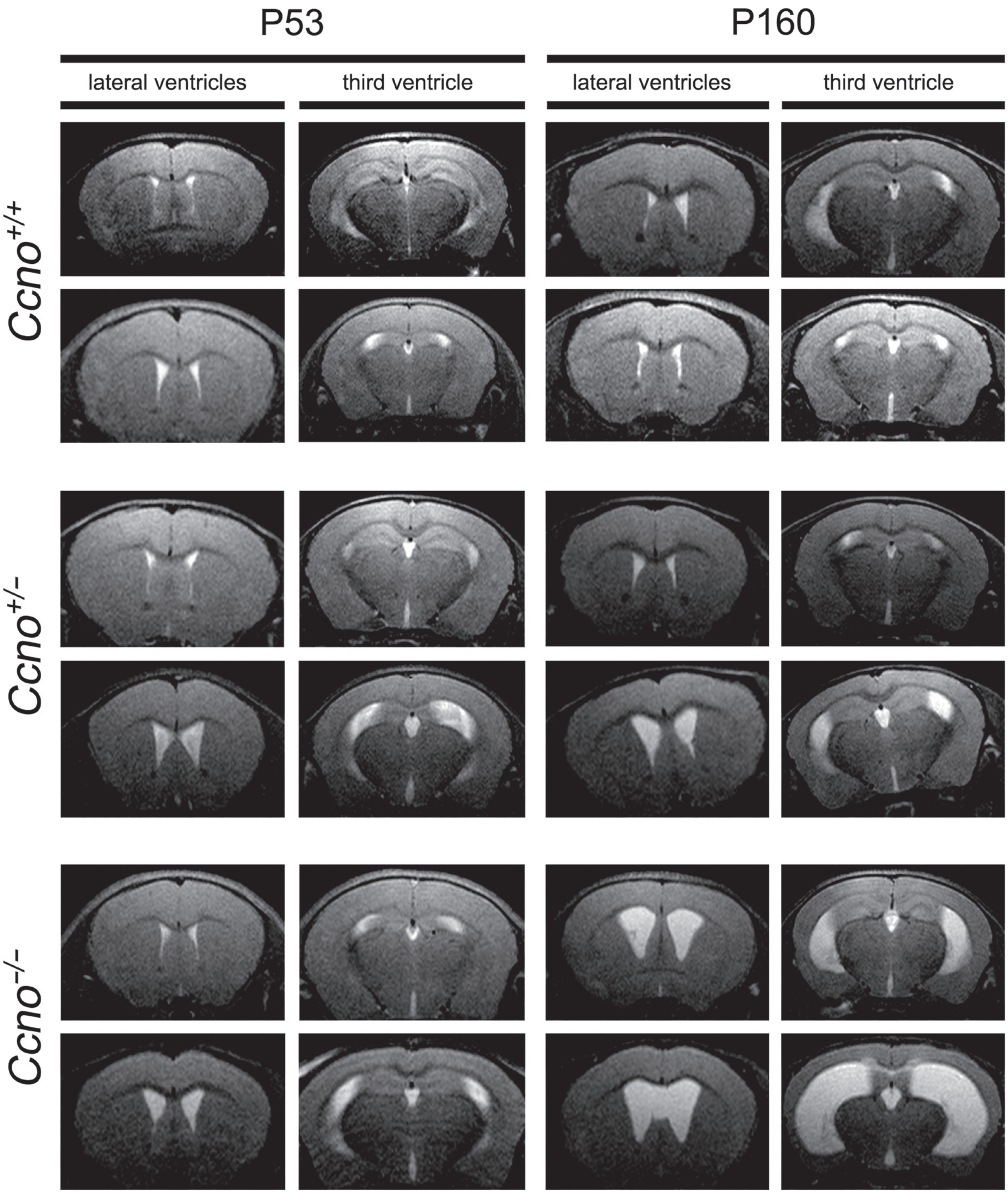

Supplementary Figure 2: MRI images at the level of the lateral ventricles or third ventricle of young (left panels, mean age 53 days) and adult (right panels, mean age 160 days) representative of mice showing low grade (upper panels) or high grade (lower panels) of hydrocephalus development. Upper panels *Ccno*$^{+/+}$ mice; middle panels *Ccno*$^{+/-}$ mice; lower panels *Ccno*$^{-/-}$ mice.

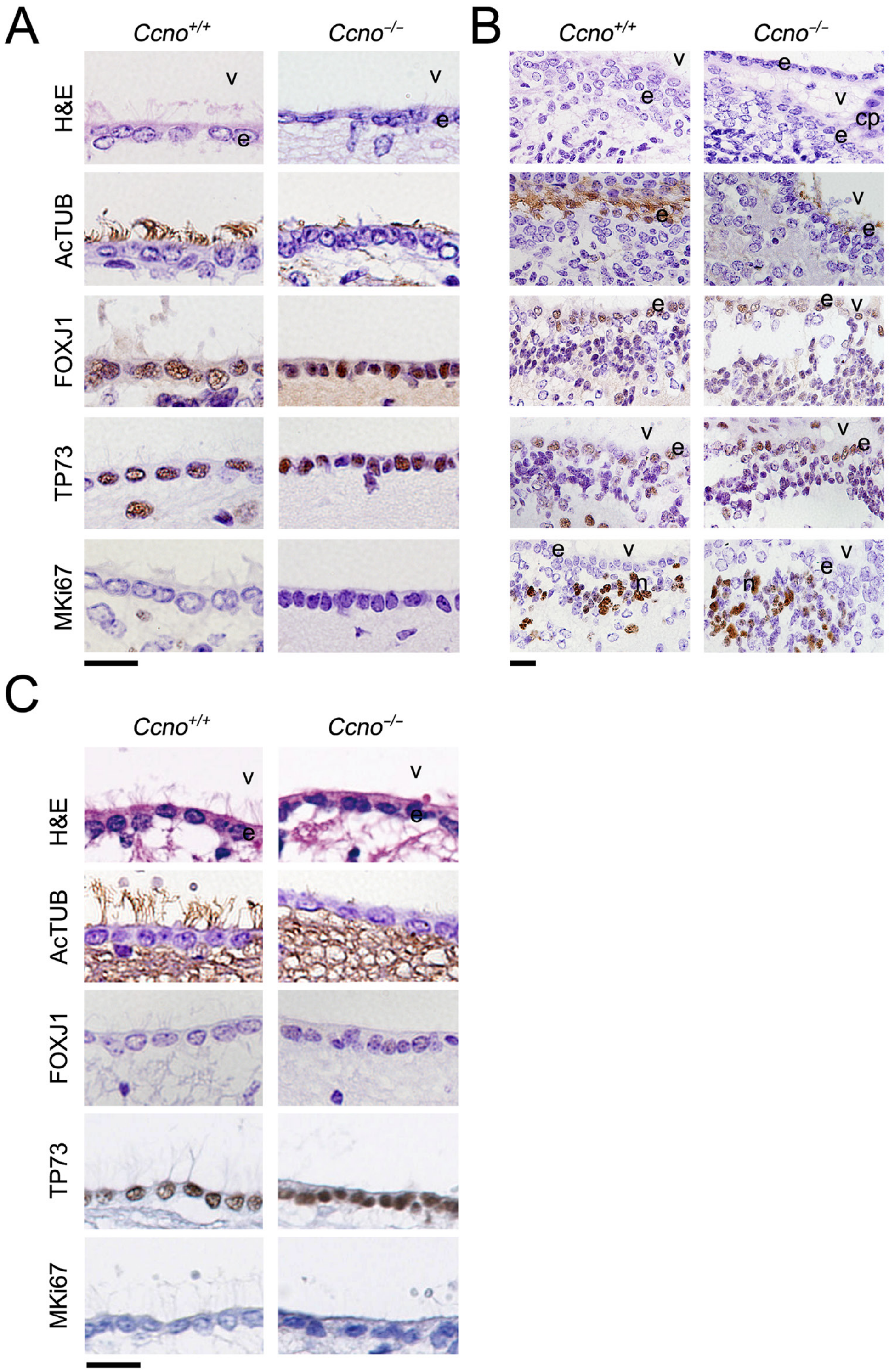

Supplementary Figure 3: Ependymal cells in the CNS of Ccno-/- mice. Brain sections from *Ccno*$^{+/+}$ and *Ccno*$^{-/-}$ mice were stained with H&E and immunohistochemistry performed with antibodies against the ciliary marker acetylated-α-Tubulin (AcTUB), the ciliated cell lineage transcription factor FOXJ1, the regulator of multiciliogenesis TP73 and the cell cycle marker MKI67. A. Ependymal cells in the ventricles of P16 mice. Scale bar, 20µm. B. Ependymal cells in the SVZ region of P16 mice. Scale bar = 20µm. C. Ependymal cells in the ventricles of P84 mice. Scale bar = 20µm. v: ventricle; e: ependyma; cp: choroid plexus; n: neuroblasts.

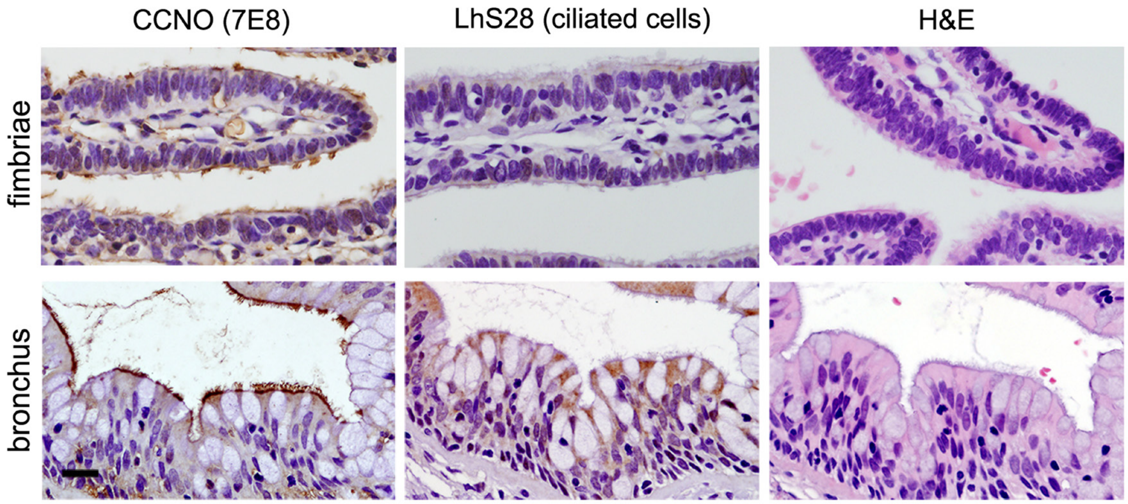

Supplementary Figure 4: Expression of CCNO in the ciliated cells of the Fallopian tube and the human bronchus. Formaldehyde-fixed, paraffin embedded 3 µm sections from biopsies from normal human Fallopian tubes (fimbriae; upper panels) and bronchi (lower panels) were obtained under permission from the MAR Biobanc and CCNO (left panels) and the ciliate cell marker LhS28 (middle panels) were detected by immunohistochemistry. The H&E staining is shown in the right panels. Scale bar = 20 µm.
